# Efficacy of PD-1/PD-L1 and LAG-3 immune checkpoint inhibitors in the treatment of patients with solid tumor

**DOI:** 10.3389/fimmu.2026.1809975

**Published:** 2026-06-16

**Authors:** Dahai Hu, Junyi Duan, Piao Xie, Jixing Li, Luyu Zhao, Bin Chen, Rongzhao Lu, Hui Tang, Xiaofei Zheng, Liangzhuo Qu

**Affiliations:** 1 Department of Sports Medicine, The First Affiliated Hospital, Guangdong Provincial Key Laboratory of Speed Capability, The Guangzhou Key Laboratory of Precision Orthopedics and Regenerative Medicine, Jinan University, Guangzhou, China; 2 Department of Gynecology, Zhuhai Clinical Medical College of Jinan University (Zhuhai People’s Hospital, The Affiliated Hospital of Beijing Institute of Technology), Zhuhai, China; 3 Department of Ophthalmology, The First Affiliated Hospital, Jinan University, Guangzhou, China; 4 Stomatology Department, The First Affiliated Hospital of Jinan University, Guangzhou, China; 5 Ultrasound Department, The First Affiliated Hospital of Jinan University, Jinan University, Guangzhou, China; 6 The Clinical Medicine Research Institute, The First Affiliated Hospital of Jinan University, Guangzhou, China; 7 Breast Surgery Department, The First Affiliated Hospital of Jinan University, Jinan University, Guangzhou, China; 8 Department of Medical Laboratory, The Fifth Affiliated Hospital of Jinan University (Heyuan Shenhe People’s Hospital), Heyuan, China

**Keywords:** efficacy, lymphocyte activation gene 3, programmed cell death ligand 1, programmed cell death protein 1, solid tumor

## Abstract

**Background:**

Lymphocyte activation gene 3 (LAG-3) inhibitor, demonstrates limited antitumor efficacy when used alone. Therefore, the clinical efficacy of combining LAG-3 with other immune checkpoint inhibitors warrants further investigation.

**Objective:**

The objective of this study is to investigate the efficacy of programmed cell death protein 1 (PD-1)/programmed cell death ligand 1 (PD-L1) and LAG-3 inhibitors in the treatment of patients with solid tumor.

**Materials and methods:**

We systematically and independently conducted a literature search using PubMed, Cochrane, and Web of Science, up to December 2025. We selected original studies investigating the efficacy of PD-1/PD-L1 combined with LAG-3 inhibitors in the treatment of solid tumors. Two authors independently extracted trial characteristics and intervention details using a predefined form. Objective response rate (ORR), disease control rate (DCR), median overall survival (OS) or progression−free survival (PFS) with their corresponding 95% confidence intervals (CIs) were used to evaluate the primary outcomes.

**Results:**

A total of six randomized controlled trials were included in the analysis. The results indicated that PD-1/PD-L1 combined with LAG-3 inhibitors significantly improved the DCR (0.66, *P* < 0.001) and ORR (0.25, *P* < 0.001) in patients with solid tumors. The median PFS of combination therapy was 3.51 months (*P* < 0.001). Subgroup analyses revealed that the Chinese population (ORR 0.4, *P* = 0.001) and individuals aged < 60 years (0.08, *P* < 0.001) derived greater benefit from PD-1/PD-L1 combined with LAG-3 inhibitor therapy. Additionally, PD-1/PD-L1 combined with LAG-3 inhibitors significantly improved the ORR in patients with nasopharyngeal carcinoma (0.24, *P* = 0.003).

**Conclusions:**

PD-1/PD-L1 combined with LAG-3 inhibitors demonstrates higher response rates, but the survival outcomes remain unclear. Further, patients with NPC, Chinese population, and individuals aged < 60 years may potentially benefit from the combination therapy.

## Introduction

In recent years, traditional treatments such as surgery, radiotherapy, and chemotherapy have remained common approaches for treating tumors, but their limitations are evident, including issues like postoperative recurrence, metastasis, and significant side effects (1, 2). However, the emergence of immunotherapy has provided a new approach to address the limitations of traditional treatments. A key advancement in immunotherapy is the development of immune checkpoint inhibitors (ICIs), which leverage the body’s own immune system to target tumors (3–6). ICIs primarily includes several types such as programmed cell death protein 1 (PD-1), programmed cell death ligand 1 (PD-L1), cytotoxic T-lymphocyte antigen 4 (CTLA-4), and lymphocyte activation gene 3 (LAG-3). Nivolumab, approved by the FDA in 2014, became the first PD-1 inhibitor used for cancer treatment, ushering in a new era of cancer immunotherapy (7). Subsequently, various PD-1 and PD-L1 inhibitors, including pembrolizumab, atezolizumab, durvalumab, and avelumab, were approved for the treatment of multiple cancers (8, 9). LAG3, composed of 503 amino acids, is an important immune checkpoint protein that plays a significant role in immune regulatory responses. LAG-3 is primarily expressed on various immune cells, such as natural killer cells, regulatory T cells, effector T cells, and activated B cells (10). Following the clinical application of CTLA-4 and PD-1/PD-L1, LAG-3 has become the third immune checkpoint receptor to be applied in clinical practice. Currently, multiple clinical trials have demonstrated the excellent therapeutic potential of LAG-3 (11–13). This progress is expected to further improve the prognosis of cancer patients and expand the field of immuno-oncology.

Currently, the combination of ICIs with traditional therapies such as chemotherapy and radiotherapy are also being explored to improve the efficacy of tumor treatment. Studies have shown that compared with monotherapy, the outcomes of combined blockade of PD-1/PD-L1 and CTLA-4 have improved (14). Additionally, research by Song et al. indicates that PD-1/PD-L1 inhibitors combined with CTLA-4 inhibitors demonstrate favorable clinical efficacy in treating colorectal cancer (15). This may be related to the fact that CTLA-4 and PD-1 target non-redundant pathways, and combining inhibitors of both could produce additive or synergistic effects (16). However, since ICIs disrupt T-cell tolerance to self-antigens, the combination of ICIs may increase the risk of immune-related adverse events (17–19). Currently, some clinical studies are attempting to combine PD-1/PD-L1 and LAG-3 for tumor treatment, but the efficacy remains somewhat controversial. Whether combining PD-1/PD-L1 and LAG-3 in immunotherapy will provide therapeutic benefits to patients with cancer requires further investigation.

The purpose of this study is to investigate the efficacy of combining PD-1/PD-L1 and LAG-3 inhibitors in treating patients with solid tumors. Furthermore, subgroup analyses based on country, tumor type, and age will be conducted to explore the factors influencing the efficacy of PD-1/PD-L1 and LAG-3 inhibitor combination therapy.

## Materials and methods

### Search strategy

This study was conducted in accordance with the Preferred Reporting Items for Systematic Reviews and Meta-Analyses (PRISMA) guidelines. The two authors (HDH and HT) systematically and independently conducted a literature search using PubMed, Cochrane, and Web of Science, up to December 2025. The search strategy included the terms ‘cancer’ OR ‘neoplasm’ OR ‘tumor’, AND ‘programmed death receptor-1 inhibitors’ OR ‘programmed death receptor ligand-1 inhibitors’ OR ‘PD-1 Inhibitors’ OR ‘PD-L1 Inhibitors’ OR ‘immunotherapy’ OR ‘immune checkpoint inhibitors’, AND ‘LAG-3 inhibitor’ OR ‘Lymphocyte activation gene 3 inhibitor’, combined with ‘clinical trial’. Additionally, the reference lists of the included studies were also reviewed as part of the process.

### Inclusion and exclusion criteria

The inclusion criteria were as follows: 1) studies investigating the impact of combining PD-1/PD-L1 and LAG-3 on the efficacy of ICI therapy.; 2) studies reporting overall survival (OS), progression-free survival (PFS), objective response rate (ORR) or disease control rate (DCR) outcomes with corresponding 95% confidence intervals (CIs); 3) observational studies or randomized controlled trials; 4) studies involving participants aged over 10 years.

The exclusion criteria are as follows: 1) studies on non-solid tumors, such as multiple myeloma, leukemia, lymphoma, etc.; 2) studies published in languages other than English; 3) studies involving non-human subjects; 4) studies in the form of reviews, meta-analyses, conference abstracts, or letters; 5) studies lacked corresponding outcome evaluations, such as OS, PFS, or ORR.

### Data extraction

Data extraction was performed independently by two researchers (HDH and HT), with any discrepancies resolved through consultation with a third researcher. The extracted data included study, year, country, sample size, gender, age, study type, tumor condition, tumor type, type of ICI, outcomes (including OS/PFS/ORR/DCR, 95% CIs), evidence grade.

### Evaluation of study quality

The Cochrane Risk of Bias Tool was used to assess the quality of the randomized controlled studies (20). This primarily includes biases arising from the randomization process, intervention measures, missing outcome data, outcome measurement, and selective reporting. Each aspect is categorized as high risk, low risk, or unclear. The studies were subsequently classified into three quality levels: weak, if three or more aspects were rated as low risk or unclear; strong, if only one aspect was rated as low risk or unclear; and moderate, for all other cases that do not fall under strong or weak.

### Data analysis

OS/PFS/ORR/DCR and the corresponding 95% CIs were used to evaluate the clinical efficacy of PD-1/PD-L1 combined with LAG-3 inhibitors in patients with solid tumors. Adjusted effect sizes were employed when possible. A random-effects model was applied for the meta-analysis of effect sizes (OS/PFS/ORR/DCR) and their corresponding 95% CIs using Stata version 15 (StataCorp LLC, College Station, TX, USA). For PFS and OS, the pooled effect measure was the median survival time (months) derived from individual studies. For ORR and DCR, pooled event rates were calculated.

Furthermore, we performed subgroup analyses based on country, tumor type, and age. The I² statistic was used to assess heterogeneity among the results. An I² > 50% indicated significant heterogeneity. Egger’s test was employed to evaluate publication bias. Sensitivity analysis was used to assess whether any single study had an evident impact on the pooled effect estimate. Finally, except for heterogeneity, a p-value of less than 0.05 was considered statistically significant.

## Result

**Figure 1:**
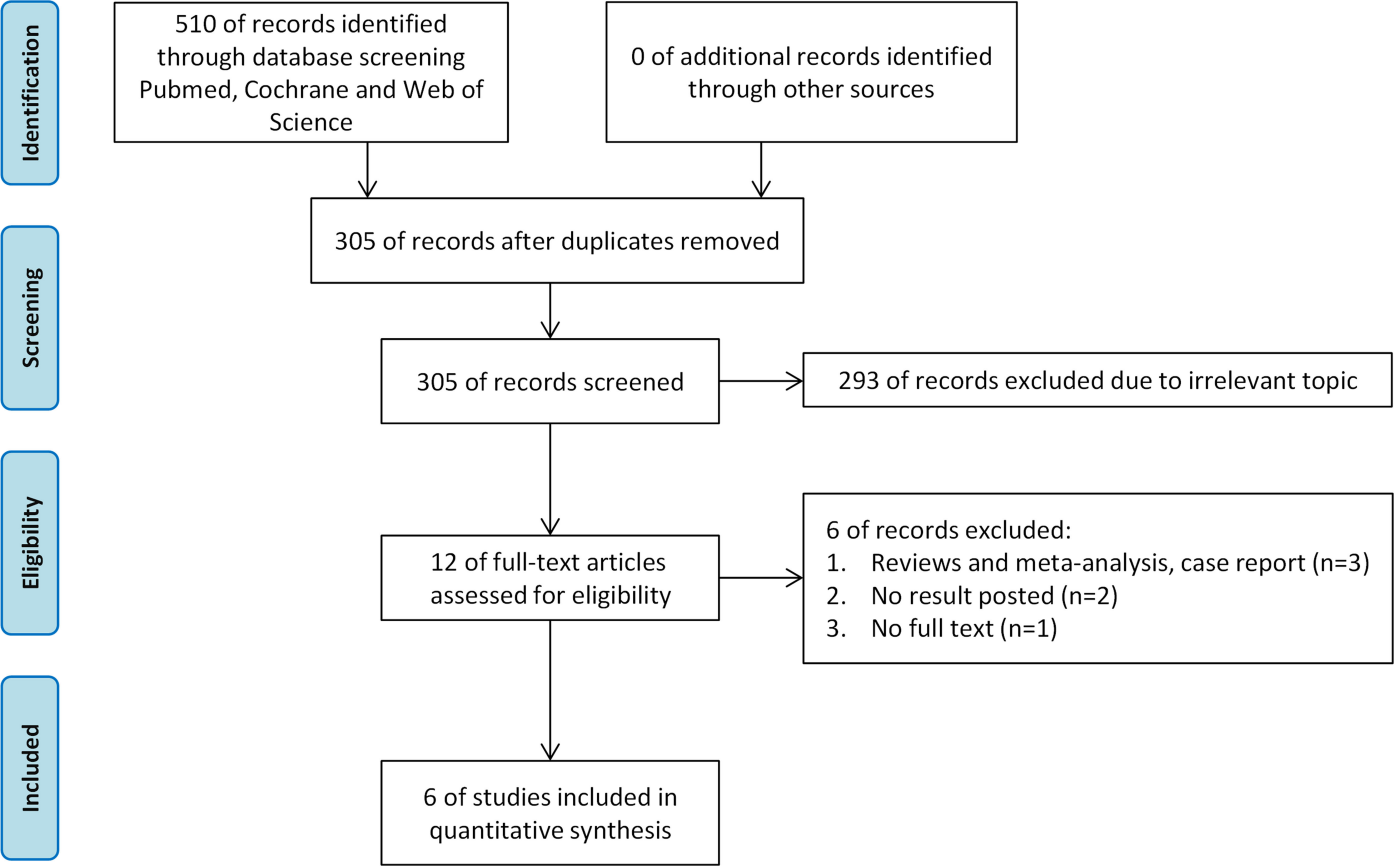
The flow chart.

Initially, a total of 510 potentially relevant studies were identified. After removing 205 duplicate records, 293 studies were excluded based on title and abstract screening, leaving 12 studies for full−text assessment (**Figure 1**). According to the inclusion and exclusion criteria, six studies were excluded, including reviews, meta−analyses, or case reports (n = 3), studies that did not report relevant outcomes (n = 2), and studies for which the full text was unavailable (n = 1). Ultimately, six randomized controlled trials (RCTs) were included in the analysis (21–26).

A total of 707 participants were included in this study. All included studies were RCTs. Among the included studies, two (33.3%) were from China and four (66.7%) were from the United States of America (**Table 1**). Regarding the years of the studies, two were conducted in 2022, one in 2023, two in 2024, and one in 2025 (**Table 1**). Furthermore, the quality of the included studies was assessed, with five (83.3%) rated as moderate and one (16.7%) as high (**Table 2**).

**Table 1:** Characteristics of included studies.

Study	Year	Country	Sample size	Gender (% men)	Age (median)	Study type	Tumor condition	Tumor type	Type of ICI
Chen (21)	2025	China	12	75.00	NR	SAT	IV	Naive NPC	LBL-007, toripalimab
Chen1			18	77.80	NR	SAT	IV	Treated NPC	LBL-007, toripalimab
Chen2			21	90.50	NR	SAT	IIIA、IIIB、IV	Naive mix	LBL-007, toripalimab
Chen3			29	69.00	NR	SAT	IV	Treated mix	LBL-007, toripalimab
Mao (22)	2024	China	28	64.30	60.5	SAT	IIIA、IIIB、IV	Mix	IBI110
Mao1			45	82.20	60	SAT	III,IV	Mix	IBI110, sintilimab
Mao2			20	95.00	63	SAT	IIIB、IIIC、IV	NSCLC	IBI110, sintilimab, chemotherapy
Mao3			17	82.40	61	SAT	IIIB、IV	GC	IBI110,sintilimab,chemotherapy
Garralda (23)	2022	USA	89	45.00	58	SAT	NR	mCRC	Favezelimab, pembrolizumab
kelly (24)	2024	USA	16	81.00	66	RCT	NR	GEC	nivolumab–relatlimab
Lin (25)	2023	USA	16	56.00	67	SAT	NR	Mesothelioma	Ieramilimab, spartalizumab
Lin2			22	68.00	57.5	SAT	NR	Melanoma	Ieramilimab, spartalizumab
Lin3			19	68.00	55	SAT	NR	RCC	Ieramilimab, spartalizumab
Lin4			14	0.00	52.5	SAT	NR	Breast cancer	Ieramilimab, spartalizumab
Tawbi (26)	2022	USA	355	59.00	63	RCT	NR	Melanoma	Nivolumab, Relatlimab

ICI, immune checkpoint inhibitors; RCT, randomized controlled trial; SAT, single-arm trial; NPC, nasopharyngeal carcinoma; NSCLC, non-small cell lung cancer; GC, gastric cancer; GEC, Gastroesophageal cancer; RCC, Renal Cell Carcinoma; NR, non report.

**Table 2:** Outcomes of the included studies and their evidence grade.

Study	Year	Outcome				Evidence grade
		PFS (median)	OS (median)	ORR	DCR	
Chen	2025	10.8(1.3-NR)	NR	0.333(0.099-0.651)	0.750(0.428-0.945)	Moderate
Chen1		2.7(1.4-4.9)	NR	0.118(0.015-0.364)	0.647(0.383-0.858)	
Chen2		3.1(1.3-5.7)	NR	0.158(0.034-0.396)	0.632(0.384-0.837)	
Chen3		1.4(1.3-1.6)	NR	0.037(0.001-0.190)	0.333(0.165-0.540)	
Mao	2024	NR	NR	NR	0.250(0.090-0.410)	Moderate
Mao1		NR	NR	NR	0.674(0.534-0.815)	
Mao2		NR	NR	0.750(0.509-0.913)	0.850(0.621-0.968)	
Mao3		12.9(3.8-15.8)	15.8(8.5-NR)	0.706(0.440-0.897)	0.941(0.713-0.999)	
Garralda	2022	2.1(1.9-2.2)	8.3(5.5-12.9)	NR	NR	Moderate
kelly	2024	NR	0.938(0.826-1.000)	NR	NR	Moderate
Lin	2023	NR	NR	0.063(0.003-0.264)	NR	Moderate
Lin2		NR	NR	0.091(0.016-0.259)	NR	
Lin3		NR	NR	0.053(0.003-0.226)	NR	
Lin4		NR	NR	0.00(0.00-0.193)	NR	
Tawbi	2022	10.1(6.4-15.7)	NR	NR	NR	High

ORR, Objective response rate; DCR, disease control rate; OS, overall survival; PFS, progression−free survival; NR, non report.

### Disease control rate

**Figure 2:**
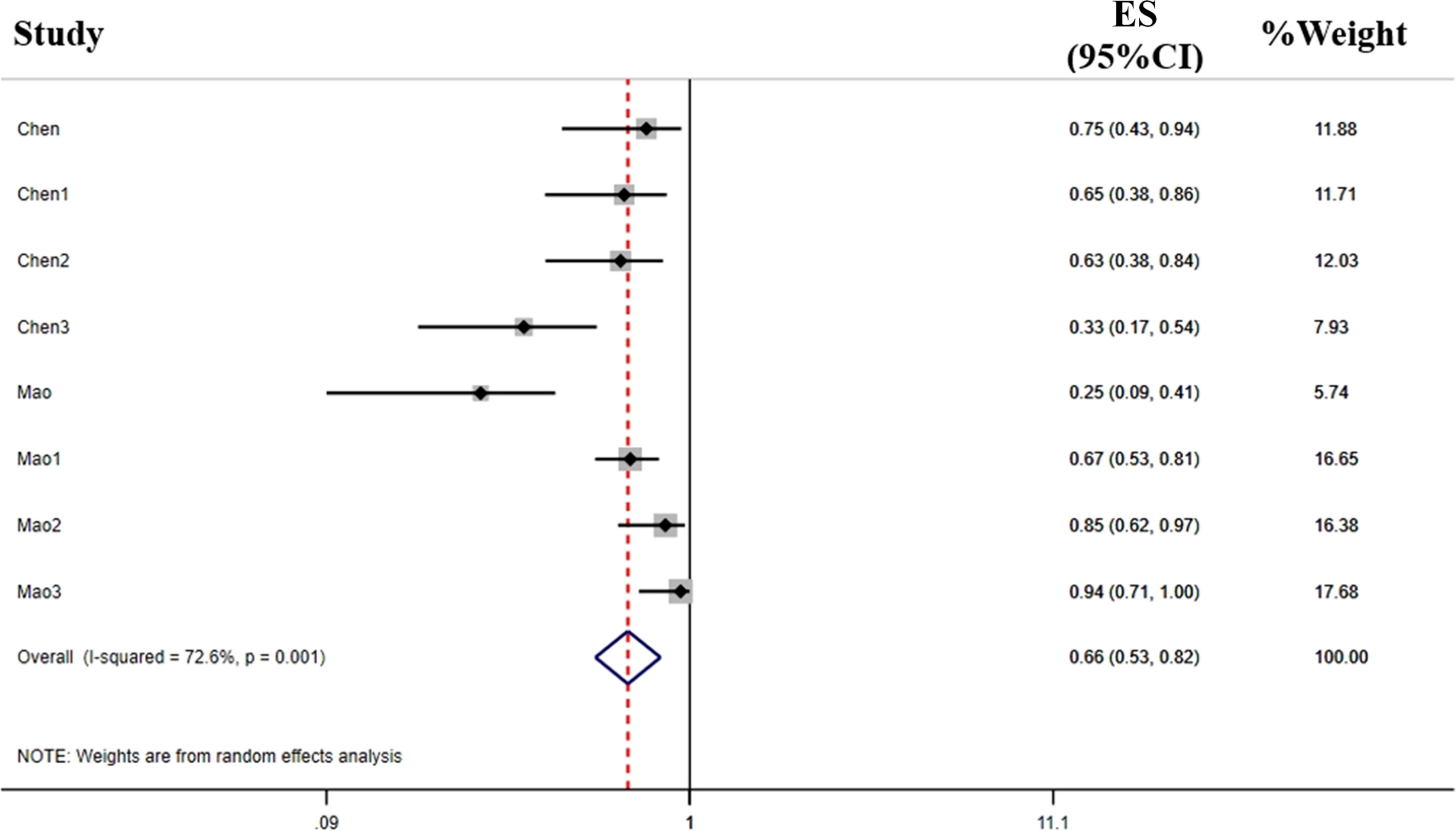
The forest plots of DCR and 95% CIs for the efficacy of PD-1/PD-L1 and LAG-3 inhibitors. DCR, disease control rate; PD-1, programmed cell death protein 1; PD-L1, programmed cell death ligand 1; LAG-3, lymphocyte activation gene 3; ES, effect size.

Compared with immunotherapy alone, PD-1/PD-L1 combined with LAG-3 inhibitors can significantly improve the DCR in patients with solid tumors (0.66 [95%CIs 0.53-0.82], *P* < 0.001) (21, 22), despite significant heterogeneity in the results (I^2^ = 72.6%, *P* = 0.001) (**Figure 2**).

### Objective response rate

**Figure 3:**
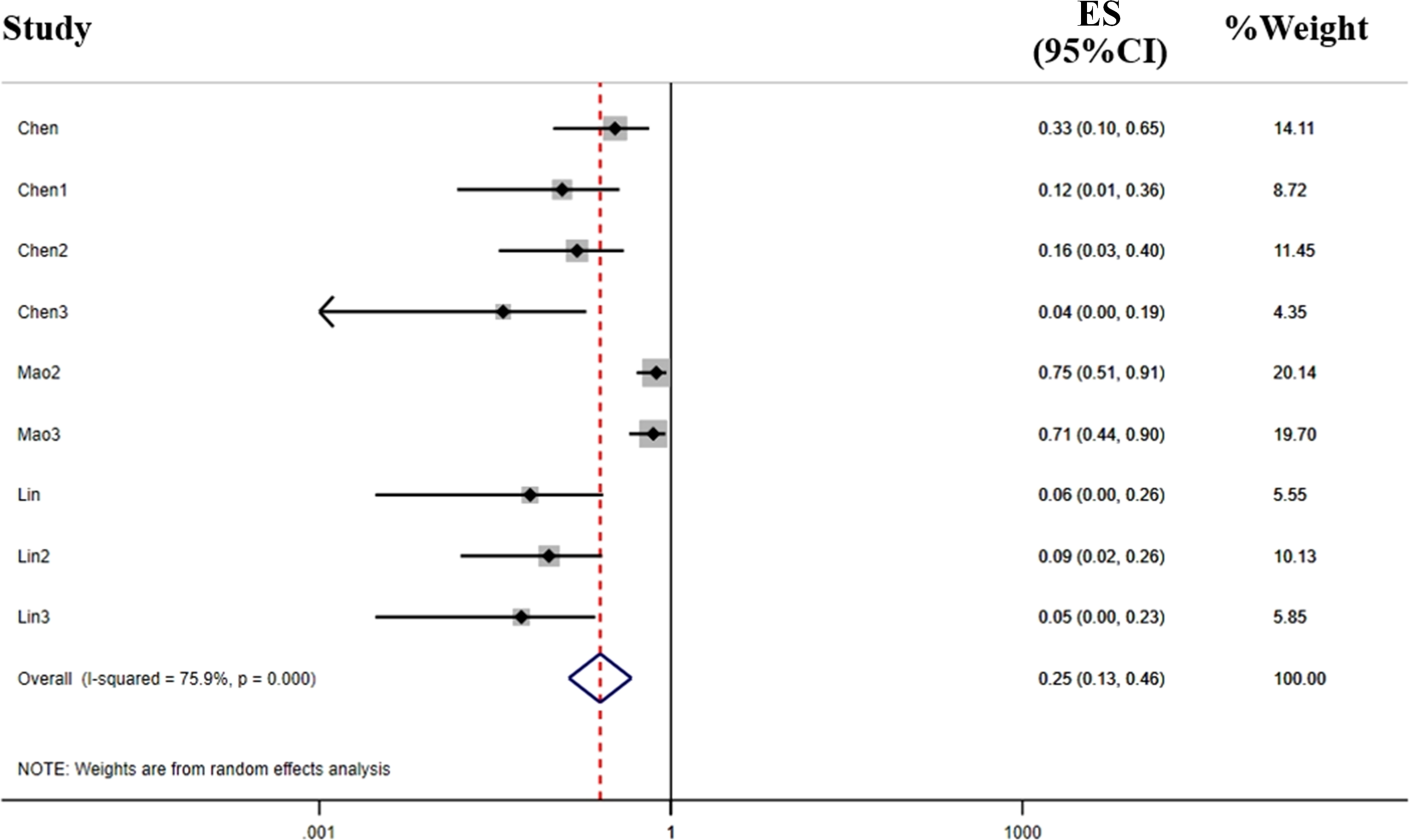
The forest plots of ORR and 95% CIs for the efficacy of PD-1/PD-L1 and LAG-3 inhibitors. ORR, objective response rate; PD-1, programmed cell death protein 1; PD-L1, programmed cell death ligand 1; LAG-3, lymphocyte activation gene 3; ES, effect size.

Furthermore, the results revealed that PD-1/PD-L1 combined with LAG-3 inhibitors significantly improves the ORR in patients with solid tumors (0.25 [95%CIs 0.13-0.46], *P* < 0.001) (21, 22, 25), with notable heterogeneity observed (I^2^ = 75.9%, *P* < 0.001) (**Figure 3**).

### Progression-free survival

**Figure 4:**
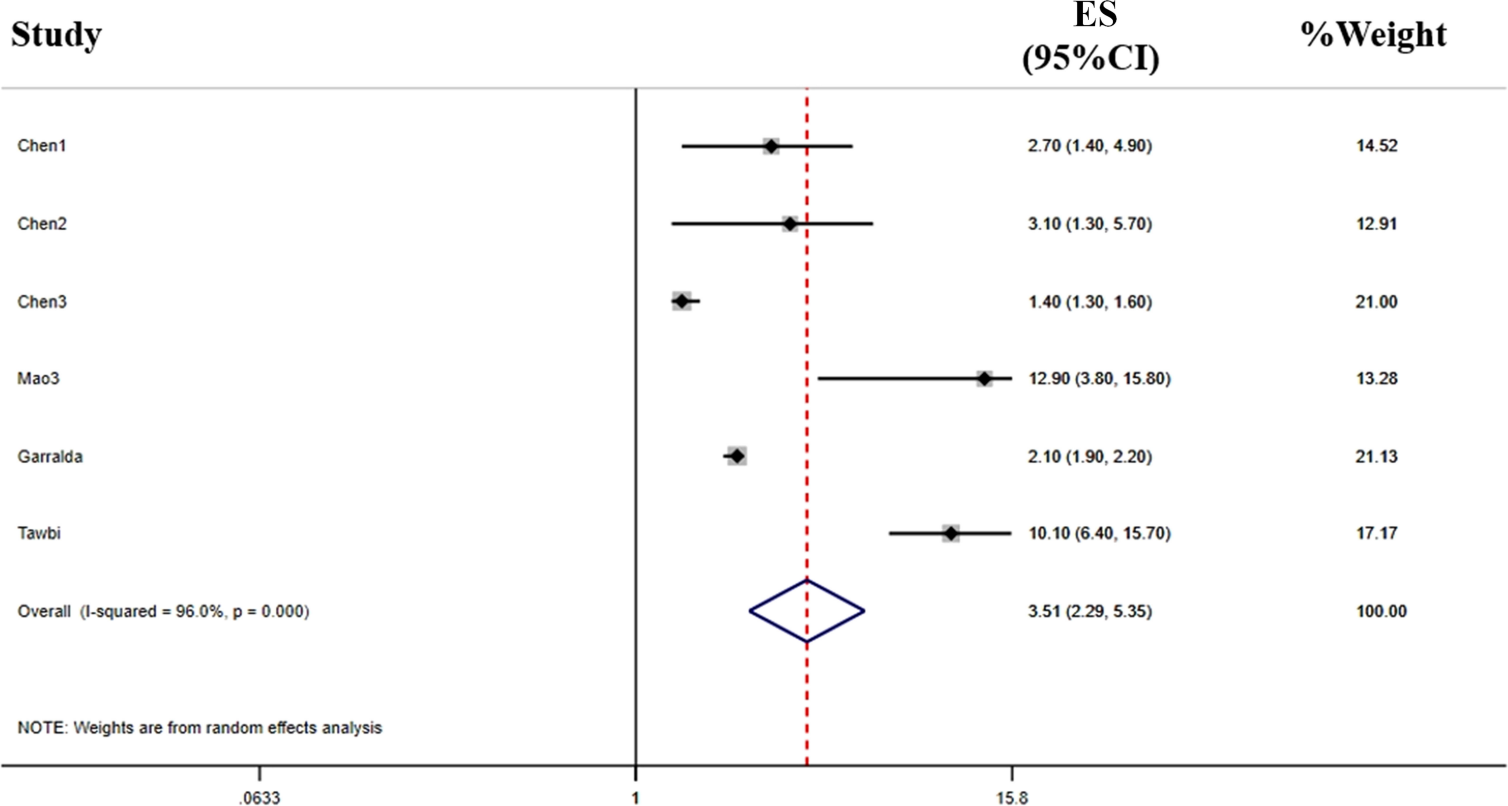
The forest plots of PFS and 95% CIs for the efficacy of PD-1/PD-L1 and LAG-3 inhibitors. PFS, progression-free survival; ES, effect size.

As show in **Figure 4**, the median PFS of PD-1/PD-L1 combined with LAG-3 inhibitors in patients with solid tumors was 3.51 months (95%CIs 2.29-5.35, *P* < 0.001) (21–23, 26), and the results exhibited significant heterogeneity (I^2^ = 96%, *P* < 0.001). Given that the majority of the included studies were single-arm trials, the pooled median PFS presented in this analysis should be interpreted as a trend reference rather than a precise predictive estimate.

### Overall survival

Furthermore, as show in **Table 1**, the median OS of PD-1/PD-L1 combined with LAG-3 inhibitors in patients with solid tumors was 8.3 months (95% CI 5.5-12.9). Moreover, a study by Kelly et al. showed that the overall 2-year OS rate for nivolumab combined with relatlimab in patients with gastroesophageal cancer was 93.8% (95% CI 82.6–100%).

### Subgroup analysis of ORR

Next, we conducted subgroup analyses based on country (China, USA), tumor type, age (≥ 60, < 60), and combination strategy. As shown in **Table 3**, PD-1/PD-L1 combined with LAG-3 inhibitors significantly improved the ORR in patients with solid tumors in both the Chinese (0.4 [95%CIs 0.23-0.7], *P* = 0.001) and American populations (0.07 [95%CIs 0.03-0.21], *P* < 0.001), with a greater benefit observed in the Chinese population compared to the American population. Then, we found that PD-1/PD-L1 combined with LAG-3 inhibitors significantly improved the ORR in patients with nasopharyngeal carcinoma (NPC) (0.24 [95%CIs 0.10-0.62], *P* = 0.003) (**Table 3**). Furthermore, PD-1/PD-L1 combined with LAG-3 inhibitors significantly increased the ORR in solid tumor patients aged < 60 years (0.08 [95%CIs 0.02-0.25], *P* < 0.001), whereas no significant improvement was observed in patients aged ≥60 years (0.67 [95%CIs 0.44-1.03], *P* = 0.065) (**Table 3**). The combined use of different types of ICIs can also affect treatment efficacy. We found that IBI110 combined with sintilimab (0.70 [95%CIs 0.49-0.99], *P* = 0.45) had the best efficacy in patients with solid tumors, followed by LBL-007 combined with toripalimab (0.19 [95%CIs 0.10-0.39], *P* < 0.001), and ieramilimab combined with spartalizumab (0.01 [95%CIs 0.002-0.10], *P* < 0.001).

**Table 3:** The subgroup analysis of ORR.

Subgroup	No. of studies	OR (95% CIs)	*P* value	Heterogeneity	
				I2 (%)	*P* value
Country					
China	6	0.4(0.23-0.70)	0.001	70.8	0.004
USA	3	0.07(0.03-0.21)	<0.001	0	0.907
Tumor type					
NPC	2	0.24(0.10-0.62)	0.003	17.1	0.272
Age					
≥60	3	0.67(0.44-1.03)	0.065	56.8	0.099
< 60	2	0.08(0.02-0.25)	<0.001	0	0.68
Combination strategy					
LBL-007+toripalimab	4	0.19(0.10-0.39)	<0.001	8.4%	0.351
IBI110+sintilimab	3	0.70(0.49-0.99)	0.45	42%	0.178
Ieramilimab+spartalizumab	5	0.01(0.002-0.101)	<0.001	76.4%	0.002

### Subgroup analysis of PFS

Furthermore, we conducted a subgroup analysis on PFS. The results revealed that the median PFS of PD-1/PD-L1 combined with LAG-3 inhibitors was 3.39 months (95%CIs 1.29-8.88, *P* = 0.013) in Chinese patients with solid tumors, and 4.53 months in US patients, although the latter was not statistically significant (95%CIs 0.97-21.11, *P* = 0.054) (**Table 4**). Furthermore, IBI110 combined with sintilimab (12.9 [95%CIs 3.8-15.8]), as well as relatlimab combined with nivolumab (10.1 [95%CIs 6.4-15.7]), demonstrated favorable median PFS in patients with solid tumors, while the combinations of LBL-007 with toripalimab (2.09 [95%CIs 1.17-3.73], *P* = 0.013) and relatlimab with nivolumab (2.10 [95%CIs 1.90-2.20]) did not show a favorable median PFS.

**Table 4:** The subgroup analysis of PFS.

Subgroup	No. of studies	OR (95% CIs)	*P* value	Heterogeneity	
				I2 (%)	*P* value
Country					
China	4	3.39(1.29-8.88)	0.013	93.2	<0.001
USA	2	4.53(0.97-21.11)	0.054	97.8	<0.001
Combination strategy					
LBL-007+toripalimab	3	2.09(1.17-3.73)	0.013	75.8%	0.016
IBI110+sintilimab	1	12.9(3.80-15.8)	NR	NR	NR
Favezelimab+pembrolizumab	1	2.10(1.90-2.20)	NR	NR	NR
Relatlimab+nivolumab	1	10.1(6.40-15.7)	NR	NR	NR

### Subgroup analysis of DCR

As shown in **Supplementary Table 1**, the results revealed that PD-1/PD-L1 combined with LAG-3 inhibitors significantly improved the DCR in Chinese patients with solid tumors (0.66 [95%CIs 0.54-0.82], *P* < 0.001). Secondly, LBL-007 combined with toripalimab (0.60 [95%CIs 0.45-0.80], *P* < 0.001), as well as IBI110 combined with sintilimab (0.82 [95%CIs 0.67-0.999], *P* = 0.049), significantly improved the DCR in patients with solid tumors.

### Sensitivity analysis

To examine the impact of individual studies on the overall results, a sensitivity analysis was performed on the effect sizes for ORR, DCR, and PFS. The findings indicated that no single study significantly influenced the final pooled effect estimate (**Supplementary Figure 1**).

### Assessment of publication bias

Egger’s test was used to assess publication bias. The effect size for PFS (*P* = 0.229) showed no significant publication bias, while ORR (*P* < 0.001) and DCR (*P* = 0.006) exhibited evidence of publication bias.

## Discussion

This study is the first to investigate the effect of PD-1/PD-L1 combined with LAG-3 inhibitors on the efficacy in patients with solid tumors. PD-1/PD-L1 combined with LAG-3 inhibitors can significantly improve DCR and ORR in patients with solid tumors. The Chinese population and individuals aged < 60 years may potentially benefit from PD-1/PD-L1 combined with LAG-3 inhibitor therapy. NPC patients receiving PD-1/PD-L1 combined with LAG-3 inhibitor treatment achieved better ORR.

LAG-3 is a recently applied immune checkpoint inhibitor in clinical practice. The results of this study indicate that its combination with PD-1/PD-L1 is beneficial for patients with solid tumors. Previous studies have also shown that LAG-3 inhibitors (e.g., IBI110) as monotherapy exhibit relatively low clinical efficacy, with only 1 out of 28 patients achieving partial response (ORR 3.6%) (22). Similar results have been observed in studies on ieramilimab and favezelimab. Therefore, LAG-3 inhibitor monotherapy appears to have limited antitumor efficacy, making the combination of LAG-3 inhibitors with other immune checkpoint inhibitors a new strategy for cancer treatment. Meanwhile, a phase I clinical study demonstrated that the combination of ieramilimab and spartalizumab achieved an ORR of 10.7% (27). In patients with advanced microsatellite stable colorectal cancer, the ORR was 6.3% for those treated with favezelimab plus pembrolizumab (23). In this study, the ORR and DCR of PD-1/PD-L1 combined with LAG-3 inhibitors were 25% and 66%, respectively, further validating the significantly enhanced efficacy of this combination therapy. The combination of PD-1/PD-L1 and LAG-3 inhibitors can amplify CD8^+^ T cell signaling and cytotoxicity while maintaining an exhausted state (28).

Additionally, this study found that the median PFS of PD-1/PD-L1 combined with LAG-3 inhibitors in patients with solid tumors was 3.51 months. One study indicated that compared with monotherapy, combination therapy leads to a higher incidence of grade 3–4 immune-related adverse events (irAEs) and an increased rate of treatment discontinuation due to toxicity, resulting in shorter actual treatment exposure and thereby diluting the efficacy benefits (29). IrAEs are also a factor that needs to be carefully considered during immunotherapy. From a clinical management standpoint, the observed PFS outcomes may also reflect treatment discontinuation or dose reductions driven by irAEs. In a nivolumab-relatlimab study for B-cell malignancies, no unexpected safety signals or dose-limiting toxicities were reported during escalation compared with anti-PD-1 monotherapy (30). Nevertheless, a considerable proportion of patients experienced irAEs of varying grades, and although most were manageable, these events remained a key determinant of treatment adherence. Importantly, clinical benefit can persist even after treatment cessation due to AEs (31). In a study combining anti-LAG-3, anti-PD-1, and chemotherapy for advanced melanoma, chemotherapy dose optimization reduced Grade≥3 AEs rates from 55.6% to 22.2%, indicating that proactive dose management enhances tolerability without compromising efficacy (32). Consequently, timely intervention and individualized dose adjustments are critical for maximizing the survival benefit of the combination therapy.

Furthermore, in some patients, simultaneous blockade of PD-1 and LAG-3 induces a “hyper-exhausted” phenotype in both peripheral and intratumoral CD8^+^ T cells, leading to a decline in effector function (29). T cells at the progenitor stage (TPEX, characterized by TCF-1^+^, PD-1^+^) and the terminal exhaustion stage (Tex-term, characterized by TIM-3^+^, TOX^+^, high PD-1 expression) play opposite roles in ICI therapy (33). Studies have shown that TPEX cells are the major T cell subset responsive to ICI therapy, and their frequency is positively correlated with long-term patient survival (33). In contrast, Tex-term cells show no significant proliferative response to ICI, and their excessive accumulation is closely associated with resistance to immunotherapy. Tumor-associated macrophages drive the differentiation of TPEX toward Tex-term through antigen presentation, leading to a decrease in the progenitor-to-terminal exhaustion ratio, thereby weakening the efficacy of ICI therapy (34). Importantly, by upregulating surface PD-L1 expression, tumor cells convert CD4^+^ T cells into immunosuppressive Foxp3^+^ regulatory T cells while attenuating the anti-tumor immune response of effector T cells, thereby enabling tumor cells to evade recognition and elimination by the immune system (35). Furthermore, the spatial heterogeneity of immune infiltration, including cell co clustering, neighborhood architecture, and tumor margin features, rather than immune cell density alone, plays a decisive role in the response to ICI therapy (36, 37). Therefore, when PD-1/PD-L1 is combined with LAG-3 inhibitors, close monitoring of AEs and T−cell exhaustion in patients is essential.

Patients with solid tumors under the age of 60 derive greater benefit from PD-1/PD-L1 combined with LAG-3 inhibitor therapy. This may be related to their immune reserve, peripheral T−cell diversity, and functional vitality compared to older individuals. Notably, the gut microbiome plays an important regulatory role in cancer immunotherapy. By producing metabolites such as short-chain fatty acids, polysaccharide A, and indole-3-carbaldehyde, the gut microbiota activates signaling pathways including Toll-like receptor 2 and the aryl hydrocarbon receptor, thereby enhancing CD8^+^ T cell activation and interferon-gamma secretion, which in turn improves the tumor microenvironment and enhances the efficacy of immunotherapy (38). Furthermore, the Chinese population appears to benefit more from this combination therapy than the American population. Previous studies have shown that the proportion of LAG-3^+^ tumor-infiltrating lymphocytes in Chinese patients with nasopharyngeal carcinoma, gastric cancer, and hepatocellular carcinoma is significantly higher than in Western populations, leading to more pronounced direct benefits due to target enrichment (21).

This study also found that NPC patients treated with PD-1/PD-L1 combined with LAG-3 inhibitors achieved an ORR of 24%. NPC exhibits highly distinct geographical distribution characteristics, with a notably high incidence in Southern China. Distinct from other solid tumors, the tumor microenvironment of NPC is often abundant in tumor-infiltrating lymphocytes. However, these lymphocytes frequently remain in a functionally exhausted state, which is primarily driven by the high-intensity chronic inflammatory stimulation induced by Epstein-Barr virus (EBV) infection (39). Furthermore, EBV also induces high expression of LAG-3 on infiltrating CD4+ and CD8+ T cells through cytokines such as IFN-γ (39). Given the limited efficacy of PD-1 inhibitor monotherapy, the combination of PD-1/PD-L1 with LAG-3 inhibitors can achieve improved therapeutic outcomes in NPC patients. Moreover, PD-1/PD-L1 combined with LAG-3 inhibitors may serve as a potential treatment option for patients with recurrent/metastatic NPC who are unsuitable for local radiotherapy or have developed radio-resistance. Radiotherapy has been shown to enhance T cell activation and tumor infiltration, while LAG-3 expression on exhausted T cells restricts the antitumor immune response induced by radiation (40). Dual PD-1/LAG-3 blockade has been demonstrated to act synergistically with radiotherapy to improve tumor control in anti-PD-1 resistant models (41). Therefore, PD-1/PD-L1 combined with LAG-3 inhibitors represents a feasible and effective therapeutic approach.

Different pairings of PD-1/PD-L1 checkpoint inhibitors with LAG-3 inhibitors yield marked differences in therapeutic efficacy. We observed that IBI110 combined with sintilimab significantly improves ORR and DCR in solid tumor patients, accompanied by extended median PFS. As a next-generation anti-LAG-3 antibody, IBI110 demonstrates higher binding affinity to LAG-3, likely exhibiting superior binding kinetics that more effectively block interactions with MHC class II molecules and fibrinogen-like protein 1 (42). Additionally, the dosage and administration frequency of the combination regimen also impact final treatment outcomes. Different drug combinations have a certain degree of heterogeneity. Therefore, regimen-specific evaluations are necessary in future studies.

PD-1/PD-L1 combined with LAG-3 inhibitors is a feasible and effective treatment strategy for solid tumors, with particularly promising prospects in Chinese populations and LAG-3-high tumor types such as NPC. Nevertheless, the increased toxicity and potential risk of T-cell hyper-exhaustion associated with combination therapy must be carefully considered. Future efforts should prioritize optimizing antibody pairings, identifying predictive biomarkers (including LAG-3 expression, EBV status, age, and ethnicity), and developing individualized dosing regimens to achieve the optimal balance between efficacy and safety.

This study has several limitations. First, the number of included studies is limited, and some pooled effect estimates exhibit a degree of publication bias and heterogeneity. Therefore, incorporating more RCTs in future research is necessary. Second, the included studies cover a relatively narrow range of tumor types, which imposes constraints on subgroup analyses. Investigating the efficacy of PD-1/PD-L1 combined with LAG-3 inhibitors across different tumor types is warranted.

## Conclusion

PD-1/PD-L1 combined with LAG-3 inhibitors demonstrates higher response rates, but the survival outcomes remain unclear. Further, patients with NPC, Chinese population, and individuals aged < 60 years may potentially benefit from the combination therapy.

## Data Availability

The original contributions presented in the study are included in the article/**Supplementary Material**. Further inquiries can be directed to the corresponding authors.
