# Supplementary material for: Efficacy of PD-1/PD-L1 and LAG-3 immune checkpoint inhibitors in the treatment of patients with solid tumor
Source: Front Immunol. 2026 Jun 16;17:1809975. doi: 10.3389/fimmu.2026.1809975 (PMC13314761; doi:10.3389/fimmu.2026.1809975)
Supplement: Supplementary file 1 [file SupplementaryFile1.docx]

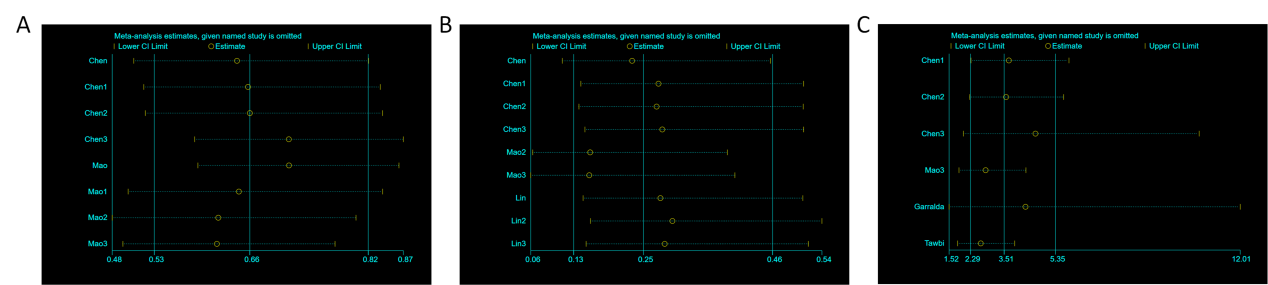


**Supplementary figure 1** The sensitivity analysis for DCR (A), ORR (B) and PFS (C).

**Supplementary table 1** The subgroup analysis of DCR.

| **Subgroup** | **No. of studies** | **OR (95% CIs)** | ***P* value** | **Heterogeneity** | |
| --- | --- | --- | --- | --- | --- |
|  |  |  |  | **I2 (%)** | ***P* value** |
| **Country** |  |  |  |  |  |
| China | 8 | 0.66(0.54-0.82) | <0.001 | 72.6 | 0.001 |
| **Combination strategy** |  |  |  |  |  |
| LBL-007+toripalimab | 4 | 0.60(0.45-0.80) | <0.001 | 41.8% | 0.161 |
| IBI110+sintilimab | 3 | 0.82(0.67-0.999) | 0.049 | 66.1% | 0.052 |
